# *N*-Cyclo­hexyl­pyrrolidine-1-carbothio­amide

**DOI:** 10.1107/S1600536812012627

**Published:** 2012-03-28

**Authors:** Yu-Feng Li

**Affiliations:** a Microscale Science Institute, Department of Chemistry and Chemical Engineering, Weifang University, Weifang 261061, People’s Republic of China

## Abstract

In the title mol­ecule, C_11_H_20_N_2_S, the five-membered ring has an envelope conformation and the cyclo­hexane ring is in a chair conformation. The N—H group is not involved in any intra- or inter­molecular inter­actions.

## Related literature

For the medicinal properties of pyrrolidine compounds, see: Yang *et al.* (1997 ▶). For related structures, see: Köhn *et al.* (2004 ▶); Li (2011 ▶).
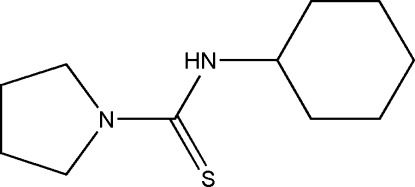


## Experimental

### 

#### Crystal data

C_11_H_20_N_2_S
*M* *_r_* = 212.35
Orthorhombic, 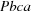

*a* = 9.3808 (19) Å
*b* = 10.925 (2) Å
*c* = 23.540 (5) Å
*V* = 2412.6 (8) Å^3^
*Z* = 8
Mo *K*α radiation
μ = 0.24 mm^−1^
*T* = 293 K
0.22 × 0.20 × 0.18 mm

#### Data collection

Bruker SMART CCD diffractometer
22078 measured reflections
2766 independent reflections
1700 reflections with *I* > 2σ(*I*)
*R*_int_ = 0.046

#### Refinement

*R*[*F* ^2^ > 2σ(*F* ^2^)] = 0.053
*wR*(*F* ^2^) = 0.190
*S* = 1.18
2766 reflections
127 parameters
H-atom parameters constrained
Δρ_max_ = 0.25 e Å^−3^
Δρ_min_ = −0.35 e Å^−3^

### 

Data collection: *SMART* (Bruker, 1997 ▶); cell refinement: *SAINT* (Bruker, 1997 ▶); data reduction: *SAINT*; program(s) used to solve structure: *SHELXS97* (Sheldrick, 2008 ▶); program(s) used to refine structure: *SHELXL97* (Sheldrick, 2008 ▶); molecular graphics: *SHELXTL* (Sheldrick, 2008 ▶); software used to prepare material for publication: *SHELXTL*.

## Supplementary Material

Crystal structure: contains datablock(s) global, I. DOI: 10.1107/S1600536812012627/lh5439sup1.cif

Structure factors: contains datablock(s) I. DOI: 10.1107/S1600536812012627/lh5439Isup2.hkl

Supplementary material file. DOI: 10.1107/S1600536812012627/lh5439Isup3.cml

Additional supplementary materials:  crystallographic information; 3D view; checkCIF report

## Figures and Tables

**Fig. 1.**
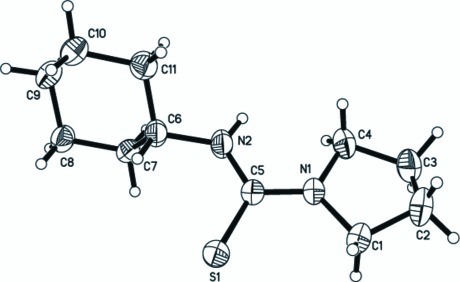
The molecular structure of the title compound showing 30% probability displacement ellipsoids.

## supplementary crystallographic information

### Comment

Pyrrolidine compounds have been shown to have medicinal properties (Yang *et al.*, 1997). The molecular structure of the title compound is shown in Fig. 1. The five-membered ring has an envelope conformation with atom C2 forming the flap. The structures related compounds have been determined (Köhn *et al.*, 2004; Li, 2011).

### Experimental

A mixture of pyrrolidine (0.6 mol), and *N*-cyclohexylmethanethioamide (0.6 mol) was stirred in refluxing ethanol (14 ml) for 4 h to afford the title compound (0.51 mol, yield 85%). Colourless blocks of the title compound were obtained by recrystallization of a solution of the title compound ethanol at room temperature.

### Refinement

H atoms were fixed geometrically and allowed to ride on their attached atoms, with C—H distances = 0.93–0.97 Å; N—H = 0.86 Å and with *U*_iso_(H) = 1.2*U*_eq_(C,*N*).

### Crystal data

**Table:**

C_11_H_20_N_2_S	*F*(000) = 928
*M**_r_* = 212.35	*D*_x_ = 1.169 Mg m^−^^3^
Orthorhombic, *P**b**c**a*	Mo *K*α radiation, λ = 0.71073 Å
Hall symbol: -P 2ac 2ab	Cell parameters from 1700 reflections
*a* = 9.3808 (19) Å	θ = 3.4–27.5°
*b* = 10.925 (2) Å	µ = 0.24 mm^−^^1^
*c* = 23.540 (5) Å	*T* = 293 K
*V* = 2412.6 (8) Å^3^	Block, colorless
*Z* = 8	0.22 × 0.20 × 0.18 mm

### Data collection

**Table:**

Bruker SMART CCD diffractometer	1700 reflections with *I* > 2σ(*I*)
Radiation source: fine-focus sealed tube	*R*_int_ = 0.046
Graphite monochromator	θ_max_ = 27.5°, θ_min_ = 3.4°
φ and ω scans	*h* = −12→12
22078 measured reflections	*k* = −13→14
2766 independent reflections	*l* = −30→30

### Refinement

**Table:**

Refinement on *F*^2^	Primary atom site location: structure-invariant direct methods
Least-squares matrix: full	Secondary atom site location: difference Fourier map
*R*[*F*^2^ > 2σ(*F*^2^)] = 0.053	Hydrogen site location: inferred from neighbouring sites
*wR*(*F*^2^) = 0.190	H-atom parameters constrained
*S* = 1.18	*w* = 1/[σ^2^(*F*_o_^2^) + (0.1021*P*)^2^] where *P* = (*F*_o_^2^ + 2*F*_c_^2^)/3
2766 reflections	(Δ/σ)_max_ < 0.001
127 parameters	Δρ_max_ = 0.25 e Å^−^^3^
0 restraints	Δρ_min_ = −0.35 e Å^−^^3^

### Special details

**Table:**

Geometry. All e.s.d.'s (except the e.s.d. in the dihedral angle between two l.s. planes) are estimated using the full covariance matrix. The cell e.s.d.'s are taken into account individually in the estimation of e.s.d.'s in distances, angles and torsion angles; correlations between e.s.d.'s in cell parameters are only used when they are defined by crystal symmetry. An approximate (isotropic) treatment of cell e.s.d.'s is used for estimating e.s.d.'s involving l.s. planes.
Refinement. Refinement of *F*^2^ against ALL reflections. The weighted *R*-factor *wR* and goodness of fit *S* are based on *F*^2^, conventional *R*-factors *R* are based on *F*, with *F* set to zero for negative *F*^2^. The threshold expression of *F*^2^ > σ(*F*^2^) is used only for calculating *R*-factors(gt) *etc*. and is not relevant to the choice of reflections for refinement. *R*-factors based on *F*^2^ are statistically about twice as large as those based on *F*, and *R*- factors based on ALL data will be even larger.

### Fractional atomic coordinates and isotropic or equivalent isotropic displacement parameters (Å^2^)

**Table:**

	*x*	*y*	*z*	*U*_iso_*/*U*_eq_	
S1	0.07717 (8)	0.34275 (5)	0.16033 (2)	0.0727 (3)	
N2	0.1756 (2)	0.57053 (15)	0.14966 (7)	0.0649 (5)	
H2A	0.2105	0.6354	0.1650	0.078*	
N1	0.1569 (2)	0.49963 (16)	0.24023 (7)	0.0625 (5)	
C5	0.1402 (2)	0.47798 (18)	0.18465 (8)	0.0533 (5)	
C6	0.1600 (2)	0.57042 (18)	0.08782 (8)	0.0565 (6)	
H6A	0.0941	0.5046	0.0773	0.068*	
C7	0.3007 (3)	0.54772 (19)	0.05863 (8)	0.0604 (6)	
H7A	0.3378	0.4686	0.0701	0.072*	
H7B	0.3687	0.6098	0.0702	0.072*	
C9	0.2197 (3)	0.6700 (2)	−0.02522 (9)	0.0637 (6)	
H9A	0.2865	0.7359	−0.0179	0.076*	
H9B	0.2032	0.6664	−0.0659	0.076*	
C10	0.0817 (2)	0.6969 (2)	0.00449 (10)	0.0647 (6)	
H10A	0.0103	0.6382	−0.0076	0.078*	
H10B	0.0490	0.7778	−0.0064	0.078*	
C11	0.0964 (2)	0.6912 (2)	0.06890 (10)	0.0657 (6)	
H11A	0.1569	0.7578	0.0817	0.079*	
H11B	0.0034	0.7015	0.0862	0.079*	
C8	0.2838 (3)	0.5505 (2)	−0.00562 (8)	0.0655 (6)	
H8A	0.3763	0.5396	−0.0233	0.079*	
H8B	0.2230	0.4834	−0.0175	0.079*	
C4	0.2130 (3)	0.6141 (2)	0.26409 (9)	0.0751 (7)	
H4A	0.1503	0.6824	0.2557	0.090*	
H4B	0.3073	0.6319	0.2493	0.090*	
C3	0.2184 (4)	0.5887 (3)	0.32761 (10)	0.0947 (10)	
H3A	0.3120	0.5596	0.3388	0.114*	
H3B	0.1960	0.6619	0.3492	0.114*	
C2	0.1095 (5)	0.4934 (3)	0.33665 (10)	0.1053 (11)	
H2B	0.0163	0.5302	0.3418	0.126*	
H2C	0.1320	0.4451	0.3700	0.126*	
C1	0.1110 (4)	0.4151 (3)	0.28475 (9)	0.0917 (9)	
H1A	0.1776	0.3477	0.2888	0.110*	
H1B	0.0169	0.3826	0.2768	0.110*	

### Atomic displacement parameters (Å^2^)

**Table:**

	*U*^11^	*U*^22^	*U*^33^	*U*^12^	*U*^13^	*U*^23^
S1	0.1120 (6)	0.0526 (4)	0.0536 (4)	−0.0127 (3)	−0.0018 (3)	−0.0005 (2)
N2	0.0950 (14)	0.0592 (11)	0.0406 (9)	−0.0187 (10)	0.0007 (8)	−0.0041 (7)
N1	0.0922 (14)	0.0560 (10)	0.0393 (9)	0.0013 (9)	0.0052 (9)	−0.0024 (7)
C5	0.0651 (13)	0.0519 (11)	0.0429 (10)	0.0033 (9)	0.0023 (9)	−0.0004 (8)
C6	0.0732 (14)	0.0557 (12)	0.0408 (10)	−0.0137 (10)	−0.0010 (9)	−0.0014 (8)
C7	0.0765 (14)	0.0565 (12)	0.0482 (11)	0.0150 (10)	−0.0002 (10)	0.0070 (9)
C9	0.0625 (14)	0.0741 (14)	0.0545 (12)	−0.0005 (10)	−0.0019 (10)	0.0182 (10)
C10	0.0600 (14)	0.0720 (14)	0.0620 (13)	0.0034 (11)	−0.0089 (10)	0.0097 (11)
C11	0.0650 (14)	0.0706 (14)	0.0617 (13)	0.0093 (11)	0.0000 (10)	−0.0031 (11)
C8	0.0774 (15)	0.0722 (14)	0.0468 (11)	0.0104 (12)	0.0073 (10)	0.0057 (10)
C4	0.1016 (19)	0.0753 (14)	0.0484 (12)	−0.0030 (14)	−0.0013 (12)	−0.0143 (11)
C3	0.139 (3)	0.096 (2)	0.0492 (13)	0.0122 (19)	−0.0130 (15)	−0.0142 (12)
C2	0.155 (3)	0.119 (3)	0.0421 (14)	0.003 (2)	0.0090 (15)	0.0021 (14)
C1	0.156 (3)	0.0742 (17)	0.0452 (13)	−0.0016 (16)	0.0119 (14)	0.0066 (11)

### Geometric parameters (Å, º)

**Table:**

S1—C5	1.691 (2)	C10—H10A	0.9700
N2—C5	1.346 (3)	C10—H10B	0.9700
N2—C6	1.463 (2)	C11—H11A	0.9700
N2—H2A	0.8600	C11—H11B	0.9700
N1—C5	1.339 (3)	C8—H8A	0.9700
N1—C1	1.462 (3)	C8—H8B	0.9700
N1—C4	1.469 (3)	C4—C3	1.522 (3)
C6—C7	1.509 (3)	C4—H4A	0.9700
C6—C11	1.515 (3)	C4—H4B	0.9700
C6—H6A	0.9800	C3—C2	1.475 (4)
C7—C8	1.521 (3)	C3—H3A	0.9700
C7—H7A	0.9700	C3—H3B	0.9700
C7—H7B	0.9700	C2—C1	1.491 (4)
C9—C10	1.500 (3)	C2—H2B	0.9700
C9—C8	1.510 (3)	C2—H2C	0.9700
C9—H9A	0.9700	C1—H1A	0.9700
C9—H9B	0.9700	C1—H1B	0.9700
C10—C11	1.524 (3)		
			
C5—N2—C6	125.71 (17)	C10—C11—H11A	109.4
C5—N2—H2A	117.1	C6—C11—H11B	109.4
C6—N2—H2A	117.1	C10—C11—H11B	109.4
C5—N1—C1	123.67 (19)	H11A—C11—H11B	108.0
C5—N1—C4	124.49 (18)	C9—C8—C7	111.29 (18)
C1—N1—C4	111.69 (18)	C9—C8—H8A	109.4
N1—C5—N2	115.87 (18)	C7—C8—H8A	109.4
N1—C5—S1	121.75 (16)	C9—C8—H8B	109.4
N2—C5—S1	122.37 (15)	C7—C8—H8B	109.4
N2—C6—C7	111.45 (17)	H8A—C8—H8B	108.0
N2—C6—C11	109.34 (16)	N1—C4—C3	103.42 (19)
C7—C6—C11	110.72 (16)	N1—C4—H4A	111.1
N2—C6—H6A	108.4	C3—C4—H4A	111.1
C7—C6—H6A	108.4	N1—C4—H4B	111.1
C11—C6—H6A	108.4	C3—C4—H4B	111.1
C6—C7—C8	110.99 (18)	H4A—C4—H4B	109.0
C6—C7—H7A	109.4	C2—C3—C4	104.3 (2)
C8—C7—H7A	109.4	C2—C3—H3A	110.9
C6—C7—H7B	109.4	C4—C3—H3A	110.9
C8—C7—H7B	109.4	C2—C3—H3B	110.9
H7A—C7—H7B	108.0	C4—C3—H3B	110.9
C10—C9—C8	111.75 (18)	H3A—C3—H3B	108.9
C10—C9—H9A	109.3	C3—C2—C1	106.3 (2)
C8—C9—H9A	109.3	C3—C2—H2B	110.5
C10—C9—H9B	109.3	C1—C2—H2B	110.5
C8—C9—H9B	109.3	C3—C2—H2C	110.5
H9A—C9—H9B	107.9	C1—C2—H2C	110.5
C9—C10—C11	112.17 (18)	H2B—C2—H2C	108.7
C9—C10—H10A	109.2	N1—C1—C2	103.2 (2)
C11—C10—H10A	109.2	N1—C1—H1A	111.1
C9—C10—H10B	109.2	C2—C1—H1A	111.1
C11—C10—H10B	109.2	N1—C1—H1B	111.1
H10A—C10—H10B	107.9	C2—C1—H1B	111.1
C6—C11—C10	111.34 (18)	H1A—C1—H1B	109.1
C6—C11—H11A	109.4		
